# Improving the Electrical Contact Performance for Amorphous Wire Magnetic Sensor by Employing MEMS Process

**DOI:** 10.3390/mi9060299

**Published:** 2018-06-14

**Authors:** Yulong Chen, Jianhua Li, Jianwen Chen, Lixin Xu

**Affiliations:** School of Mechatronical Engineering, Beijing Institute of Technology, Beijing 100081, China; 2220160063@bit.edu.cn (Y.C.); 2120160268@bit.edu.cn (J.C.); lxxu@bit.edu.cn (L.X.)

**Keywords:** amorphous alloy wire, giant magneto-impedance (GMI) magnetic sensor, micro electro mechanical systems (MEMS), solder mask

## Abstract

This paper presents a novel fabrication method for amorphous alloy wire giant magneto-impedance (GMI) magnetic sensor based on micro electro mechanical systems (MEMS) technology. In this process, negative SU-8 thick photoresist was proposed as the solder mask due to its excellent properties, such as good stability, mechanical properties, etc. The low melting temperature solder paste was used for the electrical connections with the amorphous alloy wire and the electrode pads. Compared with the conventional welding fabrication methods, the proposed micro electro mechanical systems (MEMS) process in this paper showed the advantages of good impedance consistency, and can be fabricated at a low temperature of 150 °C. The amorphous alloy wire magnetic sensor made by the conventional method and by the micro electro mechanical systems (MEMS) process were tested and compared, respectively. The minimum resistance value of the magnetic sensor made by the conventional welding method is 19.8 Ω and the maximum is 28.1 Ω. The variance of the resistance is 7.559 Ω^2^. The minimum resistance value of the magnetic sensor made by micro electro mechanical systems (MEMS) process is 20.1 Ω and the maximum is 20.5 Ω. The variance of the resistance is 0.029 Ω^2^. The test results show that the impedance consistency by micro electro mechanical systems (MEMS) process is better than that of the conventional method. The sensor sensitivity is around 150 mV/Oe and the nonlinearity is less than 0.92% F.S.

## 1. Introduction

Amorphous alloy wire has a huge and sensitive response to the small direct current (DC) magnetic field at room temperature. Therefore, it has wide application in high sensitive magnetic sensor and magnetic recording head [1]. Giant magnetoresistance effect has been found in amorphous and nanocrystal line wires [2]. Compared with the existing magnetic sensors based on Giant Magneto Resistance (GMR), Hall effect, fluxgate and superconducting quantum interference [3], amorphous alloy material show advantages of high resolution, low power consumption and fast response speed [4].

In amorphous alloy wire giant magneto-impedance (GMI) magnetic sensor fabrication, many works have been carried out. Honkura in Aichi steel company of Japan implemented the electrical connection of the amorphous alloy wire and ceramic electrodes by adoption of ultrasonic welding method [5]. Amorphous alloy wire was put into the resin-based mold, and the bias coil and feedback loops were outside the mold, then the 6-pin chip packages of sensitive components in small dimension were realized. This magneto-impedance (MI) sensor has a sensitivity of 250 mV/Oe. Zhou et al. developed a giant magneto-impedance magnetic sensor [6]. The amorphous alloy wire was fixed on a printed circuit board with the silver conducting resin and placed in a polytetrafluoroethylene (PTFE) solenoid wrapped by an enameled copper wire. Zhukov et al. studied the high-frequency giant magneto-impedance effect in Co-rich amorphous alloy wire [7]. In this study, specially designed micro strip sample holder was placed inside a sufficiently long solenoid that creates a homogeneous magnetic field, H. One end of the amorphous alloy wire was connected to the inner conductor of a coaxial line through a matched micro strip line, and the other end was connected to the ground plane. This sample holder allows measuring the samples of 6 mm length. Liu et al. connected the microwire with 24 mm long and electroplated two-terminal length of 4 mm on printed circuit board (PCB) using lead-free solder, which is composed of tin (Sn: 96.5%), silver (Ag: 3.0%), and copper (Cu: 0.5%) in wt % [8]. This method mainly reduces clustering regions with inhomogeneous distribution, disproportionate reaction and tendency of hydrogen brittleness and crack, finally achieves the stable connection by coactions of van der Waals attraction, inner diffusion and mechanical bonding. NazariNejad et al. reported a giant magneto-impedance (GMI) thin film magnetic sensor which is fabricated using conventional micro-fabrication processes [9]. Alternatively, a post-processing step was developed that allows the deposition of magnetic material to be carried out in conventional sputtering system, which circumvents the need to have special and costly sputtering systems. Chiriac et al. proposed a giant magneto-impedance (GMI) microwire array of magnetic particles for biosensor prototype [10]. The Co-Fe-Si-B glass-coated amorphous microwires were prepared by glass-coating melt-spinning method. The array consisted of 10 flexible, slightly tensioned glass coated amorphous microwires, placed as close as possible to each other inside the hollow structure of a plastic support of 50 mm × 500 μm × 500 μm. The glass cover of the micro wire ends was removed to allow electrical contact with the printed cupper leads. The glass-coated amorphous microwires were connected in “antiseries” by means of copper conductor wires so that the sense of the alternating current (AC) was the same through all of the 10 glass-coated microwires. Blanc-Béguin et al. made biological cell samples containing magnetic nanoparticles, to test and calibrate a new giant magneto-impedance (GMI) biosensor used in magnetic bio-imaging systems [11]. Co-rich amorphous uncovered microwires supplied by Unitika Ltd. were selected as sensitive magneto-impedance (MI) elements. They are made by the rotating-water spinning-method and have 10 mm in diameter, high permeability and almost zero magnetostriction coefficient. Guo et al. employed a coil-integrated giant magneto-impedance (GMI) biosensor to improve the magnetization level of magnetic particles [12]. The proposed giant magneto-impedance (GMI) biosensor is composed of symmetrical meandering Ni_77_Fe_23_/Cu/Ni_77_Fe_23_ sensing elements for biomagnetic measurement and with an integrated-3D-solenoid coil for signal amplification. The double Ni_77_Fe_23_ layers were mainly responsible for the superimposed giant magneto-impedance (GMI) effect, while the Cu layer is the pathway for the alternating current (AC). The 3D coil can be subdivided into three sections during fabrication: The bottom segment, the top segment, and the vias. Colosimo et al. presented a soft ferromagnetic glass-coated microwire to sense radio frequency (RF) and microwave energy with fiber Bragg grating (FGB) heating [13]. To create the sensor probes, the microwave-absorbing soft ferromagnetic glass-coated microwires were glued to the cladding of a commercial fiber Bragg grating (FBG). For the probes that use gold to absorb microwave energy, approximately 120 nm of the metal was sputtered onto the fiber Bragg grating’s cladding. Any temperature increase of the microwire or gold due to Joule heating was transmitted to the fiber Bragg grating (FBG) and appeared as a shift of the notch in the fiber Bragg grating’s transmission spectrum. In an earlier study by our team, we formed the micro pick-up coil of the amorphous wire giant magneto-impedance (GMI) magnetic sensor by micro electro mechanical systems (MEMS) technology [14]. In the study, the copper was electroplated at both ends of the amorphous wire for the electrical connections. Karnaushenko et al. prepared a magnetic giant magneto-impedance (GMI) stack by deposition and photolithography, and then assembled the planar device into a compact tubular structure by selectively etching the sacrificial layer [15]. To study the effects of thickness and heat treatments on giant magneto-impedance (GMI) of cobalt-coated silver wires, Jantaratana et al. electrodeposited Cobalt onto the silver wires at room temperature [16]. By keeping the dc current density constant at 150 mA/cm^2^, the layer thickness could be controlled by the deposition time. The rotating electrode technique was used to regulate the uniformity of thickness. Kammouni et al. prepared Composite metallic/ferromagnetic microtubes by electroplating Fe_20_Co_6_Ni_74_ magnetic external layer [17]. Before the electrodeposition of the magnetic layer, 3–4 μm thick copper layer was electroplated onto the previously mechanically polished surface of a CuBe cylindrical base 10 cm long. Table 1 shows the sizes and sensitivities of the above sensors.

The above amorphous alloy wire based magnetic sensors were generally fabricated by conventionally welding process. Because the welding process was not easy to control the welding area, which leads to the sensors have large size and especially poor impedance consistency. The bending stress induced during the welding process also has worse influence on the performance of giant magnetoresistive (GMI) magnetic sensors [18]. Impedance output stability of Co-rich amorphous wires depends largely on the wire-terminal-connection, which can be accomplished by composite electroplating towards a robust sensor application. The ultrasonic welding and molding technology is seriously limited by packaging accuracy and wire-connection operated process, even including large pressed stress and local crystallization resulted from ultrasonic welding [8]. In addition, the amorphous wire electroplating connection process has the disadvantages of complicated processes, and it is necessary to repeatedly perform steps such as coating photoresist and photolithography. In this paper, micro electro mechanical systems (MEMS) process was employed to fabricate amorphous alloy wire based giant magneto-impedance (GMI) magnetic sensor. SU-8 negative photoresist was used as the solder mask and low temperature solder paste (Sn_42_/Bi_58_) was used to connect the amorphous alloy wire and the electrode pads.

## 2. Device Design

**Figure 1:**
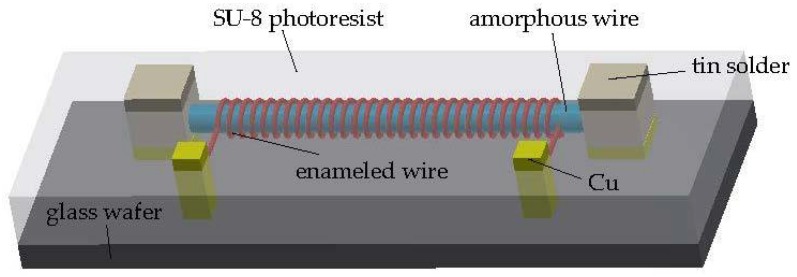
Design of the amorphous alloy wire magnetic sensor.

The device design is shown in Figure 1. The amorphous alloy wire wrapped by enameled wire is used as the sensitive element in the magnetic sensor. SU-8 negative photoresist is used as the solder mask due to its high temperature stability and good mechanical performances. The two ends of the amorphous alloy wire are electrically connected by solder. The spiral coil of enameled wire is used for extracting the response signal.

**Figure 2:**
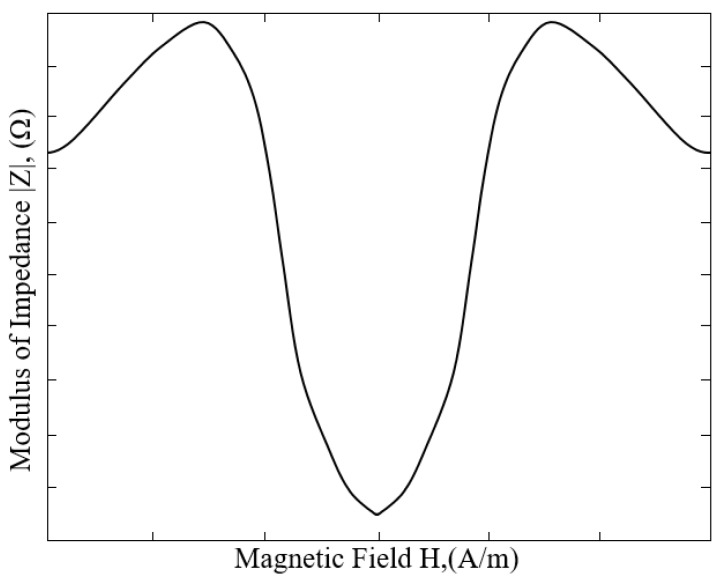
Typical curve of the GMI effect of the amorphous wire.

Circular magnetic permeability is an important factor affecting giant magneto-impedance (GMI) effect [19]. The study of different annealing treatments for amorphous wires of various compositions shows that different annealing methods can induce special magnetic anisotropy and thus improve the giant magneto-impedance (GMI) effect. The composition of the amorphous wire used in this experiment is Co_69_Fe_4.5_Ni_1.5_Si_10_B_15_, which was stress annealed and coated with glass on the surface. The amorphous wire had a length of 8 mm and a diameter of 30 μm. Typical curve of the giant magneto-impedance (GMI) effect of the amorphous wire is shown in Figure 2.

According to the law of conservation of energy, the expression of the output voltage is:

(1) $$V_{c}=\oint \vec{e}dl,$$

where the right side of the equation is the line integral of the electric field strength along the direction of the coil.

Through calculations, the output voltage can be expressed as:

(2) $$V_{c}={\vec{e}}_{\varphi }2\pi anl,$$

where ${\vec{e}}_{\varphi }$ is the component of the normal direction of the amorphous wire, *a* is the radius of the amorphous wire, *n* is the number of turns of the coil, and *l* is the length of the amorphous wire.

## 3. Device Fabrication

**Figure 3:**
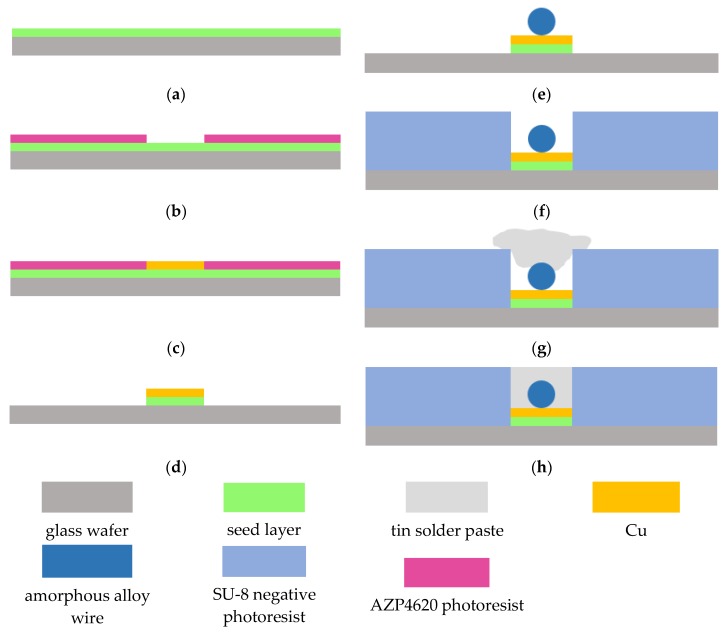
Fabrication process of the amorphous alloy wire magnetic sensor.

The fabrication process is illustrated in Figure 3. The detailed descriptions are as follows: (a) Sputter a Cr (600 Å)/Cu (2000 Å) seed layer by magnetron sputtering on the front side of the glass wafer. The sputtering conditions are: background vacuum is 1.00 × 10^−6^ torr, sputtering power of Cr is 1000 W and pressure of Ar is 5 mtorr, sputtering power of Cu is 2000 W and pressure of Ar is 8 mtorr. (b) Spin coat 5 µm AZP4620 photoresist (temperature of baking photoresist is 98 °C, time is 3 min), and then dry, expose (hard contact, the light intensity is 7.7 mw/cm^2^, the time is 170 s) and develop for 3 min. (c) Electroplate copper to form the bottom lines of the coil. Electroplating bath is sulfate. (d) Use acetone to remove the AZP4620 photoresist, and remove the seed layer. (e) Fix the amorphous alloy wire on the electroplated copper pads. (f) Spin coat 150 µm SU-8 negative photoresist (baking temperature is 98 °C, time is 3 min), and then dry, expose (hard contact, the light intensity is 7.7 mw/cm^2^, the time is 170 s) and develop for 3 min (g) Fill tin-bismuth solder paste into the patterns of the exposed areas by screen printing technology. (h) Place the substrate in a reflow oven and reflow to complete the device fabrication.

SU-8 is a negative photoresist, whose main body is the bisphenol A type epoxy resin. Firstly, bisphenol A type epoxy resin is dissolved by solution gamma-butyloracton (BGL). The volume ratio of epoxy resin to gamma-butyloracton (BGL) solution is determined according to the desired viscosity. For example, 70% epoxy resin is dissolved in 30% gamma-butyloracton (BGL) solution, and SU-8 adhesive with kinematic viscosity of 15 Pa·s can be obtained for structural studies of tens to hundreds of micrometers thick. SU-8 can be widely used in micro electro mechanical systems (MEMS) technology, due to its two important characteristics for ultra-thick structure of micro-machining. First, its low molecular weight can be dissolved in many types of solvents to form very high concentrations of up to 85% by weight solids. In addition, it has very low ultraviolet (365 nm I line) light absorption properties, which can be used as the thick structure of the manufacturing. For example, for 100 µm SU-8 photoresist, the ultraviolet (UV) light transmittance can reach 46% [20].

**Figure 4:**
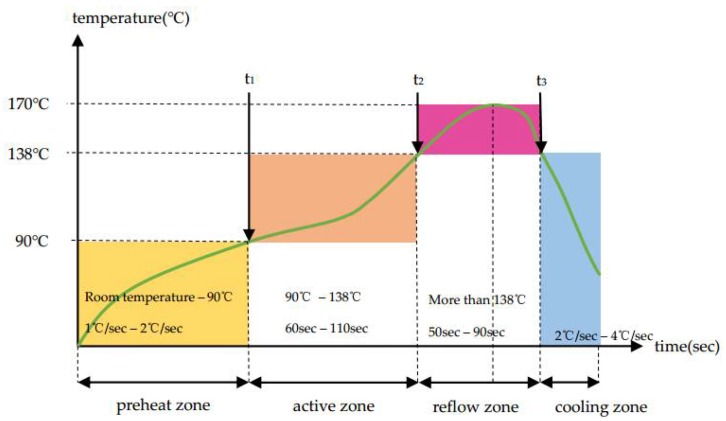
The temperature curve of Sn_42_/Bi_58_. The highest temperature of reflow zone is 170 °C, which is lower than the T_g_ of SU-8.

In this study, SU-8 is used as the tin solder paste reflow solder mask. This is because SU-8 negative photoresist shows high temperature stability. The glass transition temperature (T_g_) of the SU-8 is about 210 °C. The composition of the solder paste used in this study is Sn_42_/Bi_58_. The melting point temperature of Sn_42_/Bi_58_ is 138 °C. The reflow temperature curve is shown in Figure 4. The highest temperature is 170 °C, which is lower than the T_g_ of SU-8. Thus, SU-8 can be used as the solder mask.

## 4. Results and Discussion

### 4.1. Passive Impedance Test

**Figure 5:**
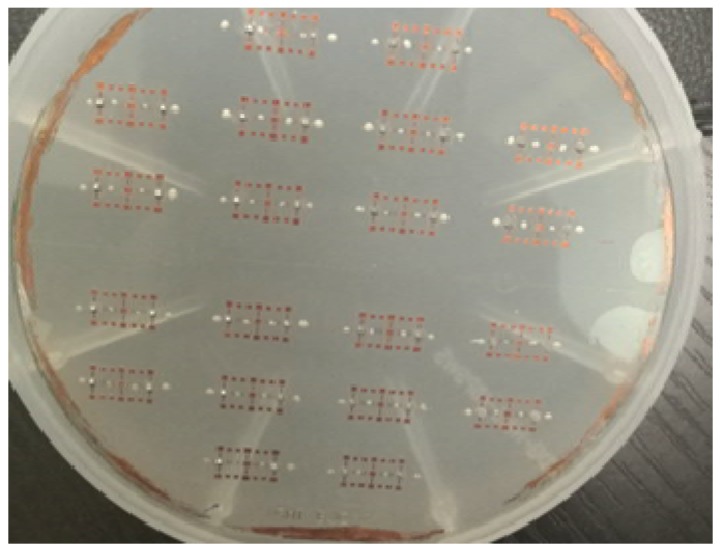
Samples of the amorphous alloy wire magnetic sensor. An array of 20 magnetic sensors is distributed on the wafer.

**Figure 6:**
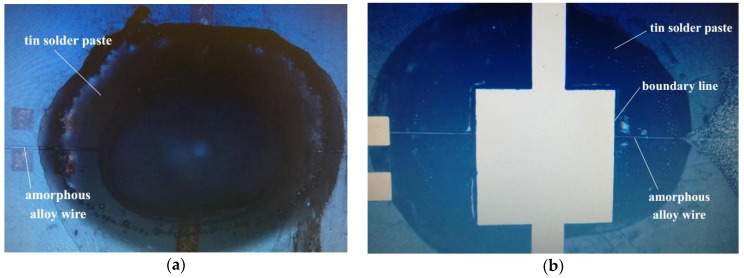
(**a**) The front image; and (**b**) the back image of the solder paste and the amorphous wire contact points under the microscope. The filled area of the tin solder paste is a square.

The fabricated samples of amorphous alloy wire based magnetic sensor are shown in Figure 5. The front image observed by the microscope is shown in Figure 6a. Although the coverage of the solder paste is still large, solder paste welding can be limited to a small area because of the SU-8 negative photoresist solder mask. The back image observed by the microscope is shown in Figure 6b. Compared with electroplating processes that require lithography and exposure, this solution adopts screen printing technology to fill the solder paste, which has the advantages of simple process.

A set of samples were fabricated by the conventional welding process for comparison. The samples were tested and the test data are shown in Table 2. The maximum resistance is 28.1 Ω and the minimum resistance is 19.8 Ω. The deviation of the samples’ resistance is 8.3 Ω and the variance is 7.559 Ω^2^, which indicates the impedance consistency is very poor.

The resistance of the samples fabricated in this study was tested. Ten samples were selected for testing, and the test data are shown in Table 3. The maximum resistance is 20.5 Ω and the minimum resistance is 20.0 Ω. The deviation of the samples’ resistance is less than 0.5 Ω and the variance is 0.029 Ω^2^, which indicates the resistance consistency is very good.

### 4.2. Active Impedance Test

**Figure 7:**
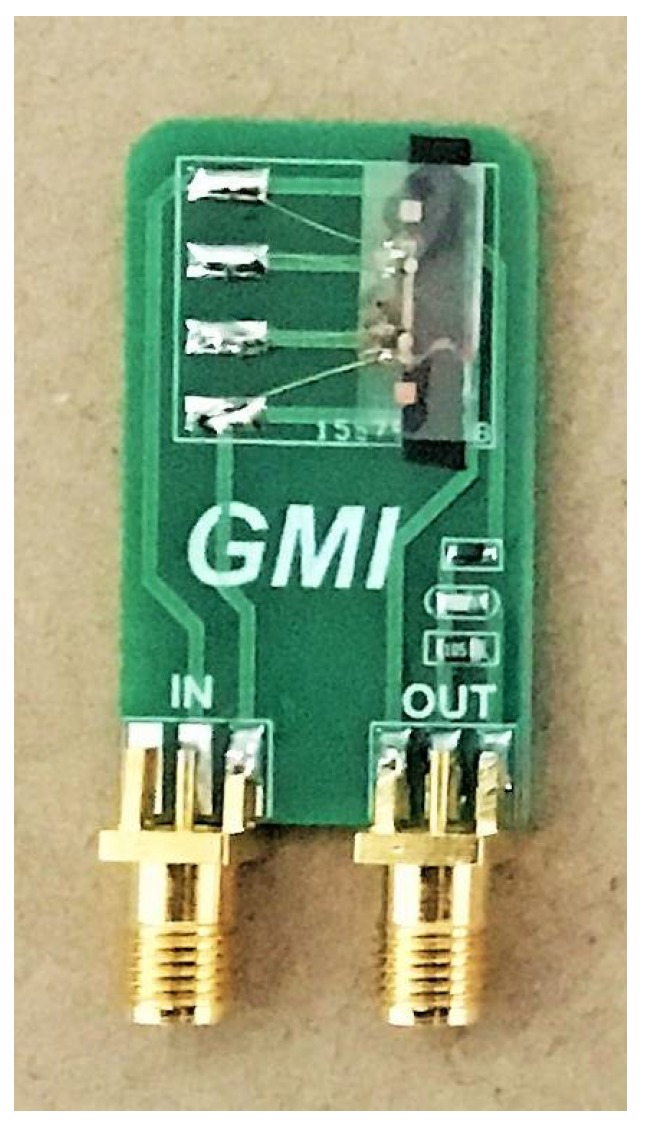
The test module of the amorphous wire alloy magnetic sensor impedance. The diode detector circuit consists of a capacitor, a resistor and a diode.

To test the sensitivity of the sensor, we connected the sample to the test module, as shown in Figure 7. In the test module, the diode detector circuit was connected to the magnetic sensor output to demodulate the magnetic field signal.

**Figure 8:**
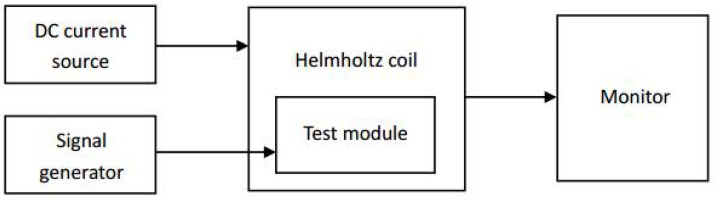
Experimental set up to measure the output of the magnetic field sensor. The outside of the Helmholtz coil is an eight-layer-metal shield that allows the sensor to be undisturbed by the external magnetic field.

As shown in Figure 8, the fabricated sensors were measured by magnetic field calibration system. The experimental setup mainly includes: electromagnetic shielding tube, Helmholtz coil, DC current source, and signal generator. When testing the sensors, they are placed in the middle of the electromagnetic shielding tube. DC current source outputs a current value every 0.05 A from −1 A to 1 A to produce a calibrated uniform magnetic field. The frequency of signal generator is set to 8 MHz, the amplitude is 5 V and the offset is 2.5 V. The output of magnetic sensors is displayed on the computer connected to the shielding tube.

**Figure 9:**
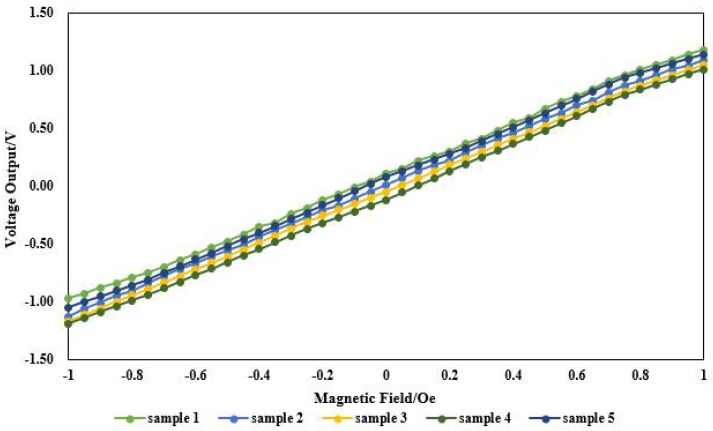
Output characteristic curve of the magnetic sensors.

To verify the feasibility of the process and product consistency, five samples were tested. The consistency curve of the sensor output is plotted in Figure 9. It can be seen in Figure 9 that the output curves of the sensors are roughly the same, indicating the good consistency of the five samples.

**Figure 10:**
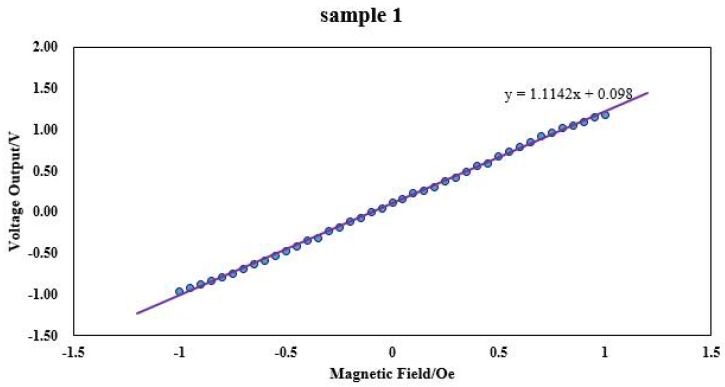
The result of linear fitting of the data of sample 1.

The data were linearly fitted and the sensitivity and linearity were calculated. The result of linear fitting of the data of sample 1 is shown in Figure 10.

Figure 10 shows the sensitivity of sensor probe is about 150 mV/Oe; the nonlinearity is less than 0.92% F.S.

### 4.3. The Frequency Characteristic of Impedance

**Figure 11:**
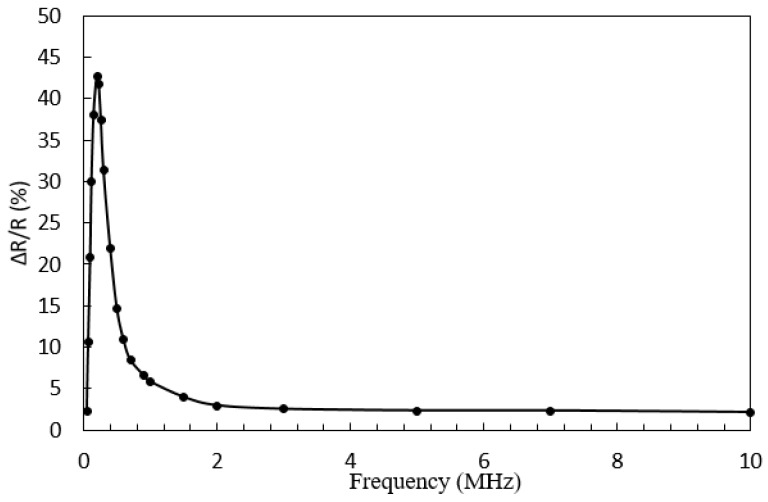
The frequency characteristic of the amorphous wire impedance.

The frequency of the excitation current strongly influences the giant magneto-impedance (GMI) characteristics. At present, the frequency range of giant magneto-impedance (GMI) materials has reached several GHz [21]. To test the frequency characteristics of amorphous wires, a digital signal generator was used to generate different frequency excitation signals and output them to both ends of the amorphous wire. The giant magneto-impedance (GMI) effect of the amorphous wire is represented by Δ*R*/*R* to simplify the experiment:

(3) $$\Delta R/R(\%)=\frac{R(H)-R(H_{\max})}{R(H_{\max})}\times 100\%$$

where *R*(*H*) is the alternating current (AC) resistance value due to external magnetic field, *R*(*H*_max_) is the saturated alternating current (AC) resistance value when the external magnetic field reaches a certain degree. The relationship between the change rate of the amorphous wire resistance and the current frequency is shown in Figure 11.

In Figure 11, we can see that, in the intermediate frequency range (5 kHz–2 MHz), the alternating current (AC) impedance of the amorphous wire changes significantly under the influence of the external magnetic field. The main reason is that the skin effect of the amorphous material is significant, and the alternating current (AC) impedance value of the sample is determined by the skin depth. Factors that affect the impedance value include the circumferential permeability of the sample, the resistivity of the material, and the alternating current (AC) drive frequency. When the resistivity of the material and the frequency of the alternating current (AC) drive are given, the permeability of the amorphous material changes significantly due to the influence of the external magnetic field, which significantly increases the current skin depth. Therefore, the real and imaginary parts of the alternating current (AC) impedance values change dramatically. In the high frequency range (>3 MHz), the movement of the domain wall is directly determined by the eddy currents. After increasing the frequency again, the dynamics becomes the dominant factor. Therefore, we can contact the Maxwell equations and the Landau–Lifshitz equations to analyze the giant magneto-impedance (GMI) effect accurately.

## 5. Conclusions

In this paper, the variance of the resistance values is used to characterize the impedance consistency of the process. The smaller the variance value is, the better the impedance consistency is. The variance of the samples produced by the traditional welding method is 7.229 Ω^2^, and that of the samples produced by micro electro mechanical systems (MEMS) technology is 0.029 Ω^2^. Test results show that the impedance consistency of the amorphous alloy wire magnetic sensor processed by micro electro mechanical systems (MEMS) process has been greatly improved. The sensitivity of sensor probe is abort 150 mV/Oe, the nonlinearity is less than 0.92% F.S. This method electroplates the ends of the amorphous alloy wire on the electrode plate, which overcomes the shortcomings of traditional welding and reduces the volume of the sensor. Besides, this process simplifies the electroplating process of connecting the amorphous wire and the copper pads and achieves a more efficient electrical connection. This method makes mass production of amorphous alloy wire magnetic sensor possible.

## Figures and Tables

**Table 1:** Summary of various amorphous wire sensors.

Name	Size	Sensitivity
GMI Thin Film Magnetic Sensor [9]	Meander Type Structures: 1 mm × 0.5 mm; Straight Wire Structures: the Length of Straight Wire is 10 mm	the Impedance in 200 kHz and 500 kHz is 136% and 145%
Coil-Integrated GMI Biosystems [10]	7.5 mm × 6.5 mm	3 ng/mL for Streptavidin-Coupled Dynabeads and 0.2 ng/mL for Alpha Fetoprotein (AFP)
Amorphous Wire Type MI Sensors for Automobile Use [5]	4 mm × 3 mm × 2 mm	250 mV/Oe
Soft Ferromagnetic Glass-Coated Microwires [11]	Wire Diameter is 14 µm, Wire Length is 3 cm	~10 times at f = 3.25 GHz Relative to the Perturbation of the Microwave Field
Operating Point Self-Regulator for GMI Magnetic Sensor [6]	Wire Diameter is 30 µm, Wire Length is 10 mm, Solenoids Length is 50 mm	0.0034 V/nT in the Range of 0–2 µT

**Table 2:** The resistance of the conventional welded amorphous wire magnetic sensors.

Num	Resistance Value/Ω	Average Value/Ω	Deviation/Ω	Variance/Ω^2^
1	23.6	24.71	8.3	7.559
2	24.5			
3	28.1			
4	25.7			
5	19.8			
6	27.5			
7	23.9			
8	25.3			
9	21.1			
10	27.6			

**Table 3:** The resistance of the new amorphous wire magnetic sensors.

Num	Resistance Value/Ω	Average Value/Ω	Deviation/Ω	Variance/Ω^2^
1	20.4	20.26	0.5	0.029
2	20.5			
3	20.2			
4	20.1			
5	20.5			
6	20.1			
7	20.3			
8	20.0			
9	20.3			
10	20.2			
